# Fracture Nonunion Treated with Low-Intensity Pulsed Ultrasound and Monitored with Ultrasonography: A Feasibility Study

**DOI:** 10.1155/2021/8834795

**Published:** 2021-01-25

**Authors:** Peizhen Zhang, Pengdong Li, Shihai Liao, Xuan Li, Wufan Chen, Xiaoyun Wang, Qing Wang

**Affiliations:** ^1^School of Biomedical Engineering, Guangdong Provincial Key Laboratory of Medical Image Processing, Southern Medical University, Guangzhou 510515, China; ^2^Guangdong Work Injury Rehabilitation Center, Guangzhou 510440, China; ^3^Nanfang Hospital, Southern Medical University, Guangzhou 510515, China

## Abstract

The positive effect of low-intensity pulsed ultrasound (LIPUS) on bone fracture healing has been proved. However, during the period of LIPUS therapy, it is undetermined whether LIPUS promotes the formation of heterotopic ossification (HO), which usually occurs in muscle tissues after trauma such as bone fracture and spinal cord injury. Here, we used 6-week LIPUS therapy in a 42-year-old Chinese male patient with a fracture nonunion in combination with ultrasonography for monitoring fracture healing and HO formation. After the LIPUS therapy, the mineralized bone formation in the area of defect of the distal tibia was presented in an ultrasound image, which was consistent with the outcome of plain radiography showing callus formation and the blurred fracture line in the area exposed to LIPUS. In addition, ultrasound images revealed no evidence of HO development within soft tissues during the period of LIPUS therapy. This study suggests that ultrasonography is a potential tool to guarantee the performance of LIPUS therapy with monitoring HO formation. Easy to use, the integration of the handheld ultrasound scanner and the ultrasonic therapeutic apparatus is entirely dedicated to help orthopedists make high-quality care and diagnosis.

## 1. Introduction

With the increasing aging population and the rapid development of transportation and construction industry, trauma usually occurs and has become one of the major health problems. However, the prognosis of trauma such as bone fracture and spinal cord injury is sometimes poor. For example, the incomplete fracture healing along with a lack of radiological sign of bony continuity over three consecutive months after the initial rehabilitation treatment is diagnosed as nonunion [1, 2], which may not heal without any intervention [3]. The “gold standard” for the nonunion treatment is surgical intervention, usually including open surgical debridement of the nonunion site and application of internal or external fixation, in most cases with bone grafting [4]. The surgical intervention of nonunion is aimed at providing adequate mechanical stability and osteogenic environment to promote healing [5]. The success rate ranges from 68% to 96% varying by nonunion site and surgery type [4]. However, the technical difficulty and the risk of complications limit the use of the surgical intervention in nonunion [2, 5, 6].

Previous studies demonstrated the effectiveness of fracture healing using low-intensity pulsed ultrasound (LIPUS). Kristiansen et al. reported a 38% reduction in the time of radiographic healing by the use of LIPUS to treat fractures [7]. In an animal model, LIPUS accelerated mature callus formation and the recovery of torsional stiffness and maximum torque [8]. Other studies investigated the mechanism of acceleration of fracture healing using LIPUS. Rawool et al. revealed that LIPUS might promote angiogenesis and increase blood supply in the tissues around the fracture [9]. It was also found that LIPUS was able to enhance adenylate cyclase activity, to transform growth factor-*β* synthesis in osteoblasts [10], and to alter aggrecan gene expression to increase proteoglycan formation in chondrocytes [11, 12]. Therefore, LIPUS can be an effective intervention applied in the treatment of fresh fractures and nonunions, which is approved by the U.S. Food and Drug Administration (FDA).

However, whether LIPUS promotes the formation of heterotopic ossification (HO) is still uncertain. HO is defined as pathological osseous tissue in the periarticular soft tissues after neurologic injury, orthopedic interventions, muscular injury, and fracture. Unlike tissue calcification due to calcium deposition, the formation of HO, a complex and dynamic developmental process, is regulated by the neuroendocrine system [13]. Wijdicks et al. demonstrated the effect of LIPUS on ectopic bone formation induced by rhBMP-2 [14]. Therefore, it is necessary to avoid HO formation during the fracture healing process with the use of LIPUS. If HO occurs, the orthopedist will properly adjust the rehabilitation treatment. However, the early and accurate diagnosis of HO is challenging.

In clinic, orthopedists usually diagnose HO by palpation and plain radiography during the rehabilitation of patients with trauma. Additionally, ultrasonography has been applied to diagnose HO. Some studies indicated that ultrasonography distinguished mature HO from the surrounding soft tissue with high specificity and effectively detected the immature HO [15–18]. Ultrasonography is useful to visualize HO lesion with several advantages, including nonionizing radiation, bedside real-time monitoring, repeatable detection, relatively simple operation, and low cost. A previous study demonstrated that the visualization of HO using ultrasonography revealed the development of HO in the muscle tissues around the injured joints [19]. Ultrasonography has potentials to provide guidance for orthopedists to make individualized rehabilitation therapy.

Therefore, in this study, we present a case with the nonunion after comminuted fracture with an aim to investigate the positive effect of LIPUS therapy on nonunion and to monitor HO occurrence that may be stimulated by LIPUS. Both radiographic and ultrasonographic outcomes suggest that LIPUS facilitates fracture repair without HO formation. In comparison with plain radiography, ultrasonography is a useful bedside tool for monitoring HO occurrence during the process of LIPUS therapy.

## 2. Materials and Methods

### 2.1. Statement of Informed Consent

This study was approved by the Ethics Committee of the Guangdong Work Injury Rehabilitation Center, Guangzhou, China. The patient provided his written informed consent to participate in this study.

### 2.2. Patient and Initial Therapy

A 42-year-old Chinese male patient with the functional limitation and pain of the right lower limb was admitted to the rehabilitation center, approximately 1 month after his plate internal fixation operation for comminuted fracture of his left calcaneus, right tibia, and fibula performed at another hospital. The physical examination revealed a poor healing of surgical incision of 12 cm with eschar on the anterior side of the right tibia. No exudate was found but the skin surrounding the surgical incision was red and rigid. The range of motion of the left ankle, bilateral hip, and knee was normal, whereas the swollen right ankle had the limited range of motion (plantarflexion: 5°, dorsiflexion: 0°). The blood test showed the absence of abnormalities.

**Figure 1:**
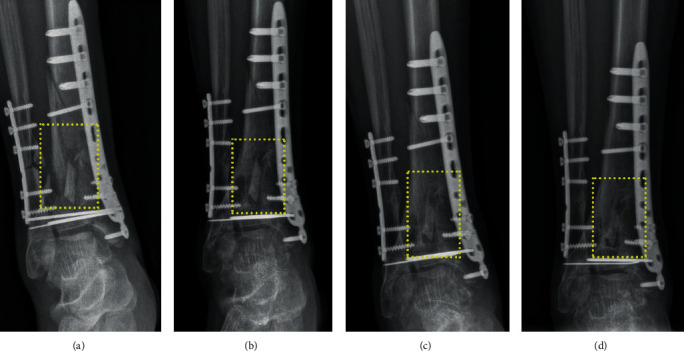
Plain radiographs of the right crus taken at the time when the patient was (a) admitted to the rehabilitation center, (b) before LIPUS therapy, (c) 1 week after the end of LIPUS therapy, and (d) 2 months after the end of LIPUS. The yellow dotted line box indicates the right distal tibial comminuted fracture region.

The patient was hospitalized. The plain radiography revealed a distinct fracture line in the right distal tibia, distal fibula surrounded with free bone fragments, and the fixations of fractures (Figure 1(a)). Then, kinesitherapy and occupational therapy were performed to improve his lower limb function and the range of ankle motion. Meanwhile, drug therapy, transcutaneous electrical nerve stimulation, and medium-frequency electrical stimulation were applied to promote blood circulation and to reduce inflammation and pain.

After approximately three and half months, the kirschner wire in the right medial malleolus was removed and the surgical debridement of the right crus was performed under local anesthesia. The aforementioned therapy measures continued. When the 5-month treatment was completed, the plain radiography was taken again to reveal the poor condition of fracture healing.  Figure 1(b) clearly shows the defect still at the right distal tibial fracture area. The distal tibial fracture was then diagnosed as nonunion because it occurred 6 months after the fracture. Therefore, LIPUS therapy was suggested to perform LIPUS to accelerate the fracture healing.

### 2.3. LIPUS Therapy and Ultrasound Monitoring

An ultrasonic system (SXUltrasonic, Shenzhen, China) for accelerating fracture healing was specially designed for the LIPUS therapy in this study. The acoustic parameters including the output power, duty cycle, pulse width, and pulse repetition frequency (PRF) were adjustable. In this study, the ultrasound transducer with a central frequency of 1.5 MHz generated ultrasound waves with a 200 *μ*s pulse width, a 20% duty cycle, a 1 kHz PRF, and a power of 30 mW/cm^2^. The handheld transducer was placed on the site of the right anterior tibial fracture with enough coupling gel interposed between the transducer and the skin. The bedside LIPUS therapy performed by one investigator would last for 20 minutes and 5 times a week for 6 weeks. During the therapy, the patient should keep his right lower limb immobilized. Besides the LIPUS therapy, the aforementioned kinesitherapy and occupational treatments were performed as well.

**Figure 2:**
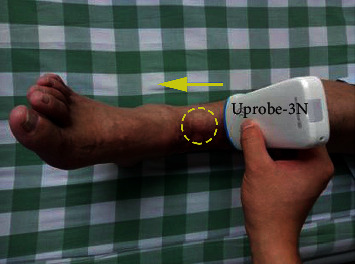
Ultrasound scanning of the right distal tibia using Uprobe-3N, a wireless ultrasound probe. The arrow indicates the scanning orientation. The dotted ellipse presents the site of LIPUS therapy.

Due to the fracture site neared entheses of extensor tendon and ankle joint, where HO might easily occur, it was necessary to monitor the LIPUS-treated bone healing process and to avoid HO formation. The ultrasound monitoring was performed once per week. A wireless ultrasound probe (Uprobe-3N, linear transducer, SonoStar, Guangzhou, China) with a fixed central frequency of 10 MHz was placed perpendicular to the distal tibia and scanned along the long axis of tibia (Figure 2). The enough coupling gel was interposed between the transducer and the skin.

## 3. Results of Diagnostic Assessment

**Figure 3:**
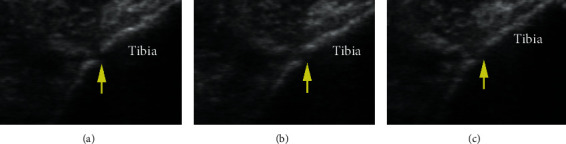
Ultrasound images at the right distal tibia examined, respectively, (a) 2 weeks, (b) 4 weeks, and (c) 6 weeks after the commence of LIPUS therapy. The arrow indicates the defect of the tibia.

As shown in Figure 3, there was a distinct defect of the right distal tibia (Figure 3(a)) 2 weeks after LIPUS therapy, and the gap became poorly demarcated with the therapy time increasing (at 4 weeks after LIPUS therapy) (Figure 3(b)). After the 6-week LIPUS therapy, the mineralized bone formation in the area of defect of the right distal tibia was observed in an ultrasound image (Figure 3(c)). Meanwhile, during the LIPUS therapy, ultrasound monitoring showed no evidence of calcification lesion within soft tissue.

The ultrasonographic outcomes were consistent with those in plain radiography (Figure 1).  Figure 1(c) shows callus formation and the blurred fracture line in the same area exposed to LIPUS. Two weeks after the end of LIPUS therapy, the patient left the hospital to recuperate at home. At 2 months after the end of LIPUS therapy, the patient went to the rehabilitation center for reexamination and presented symptoms relief of pain, swelling, and motion limitation, but mild soreness of ankle joint after a long walk. The plain radiography showed the callus formation in most of the fracture area (Figure 1(d)), and traumatic arthritis was diagnosed at the ankle joint.

## 4. Discussion

In comparison with the surgical interventions in the treatment of nonunion, LIPUS tends to be a relatively safe, noninvasive, and inexpensive method with fewer potential complications. LIPUS may be suggested when the conservative treatment is required or the surgical operations are of high risk [2, 5]. Gebauer et al. reported that the healing rate of nonunion treatment by LIPUS could reach up to 85% similar to that of the surgical methods [4]. The radiographic and ultrasonographic outcomes of this study revealed signs of progressive bone repair and suggested that LIPUS promoted fracture union.

Lower limbs support the human body and participate in the completion of walking, running, bouncing, and other movements. Therefore, the musculoskeletal system of lower limbs usually experiences mechanical force and is influenced by the mechanical environment. After long-term uselessness of the muscles and bones, atrophy, osteoporosis, and dysfunction tend to occur. Long-term immobilization of a patient with fracture contributes to the lack of mechanical force of the injury site; ultrasonic waves however produce mechanical force to improve the mechanical environment. The improvement of the mechanical environment may stimulate endochondral ossification on which one mechanism of fracture healing is based [20, 21]. However, a previous study reported that HO similarly occurred based on endochondral ossification [13]. Therefore, LIPUS may induce the formation of HO by enhancing endochondral ossification. During the process of LIPUS therapy, it needs ultrasonography to monitor HO formation.

The patient involved in this study suffered distal tibial comminuted fracture with a worse prognosis (dysfunctions of right crus, swelling, inflammation, and pain). Although he underwent 5-month kinesitherapy and drug treatment, the fracture did not heal and thus needed an intervention. LIPUS therapy is noninvasive but with one potential complication of HO occurrence because of the fracture site near entheses of extensor tendon and ankle joint. Ultrasonography was applied to monitor the fracture healing and HO occurrence. If any indications present the potential of HO in soft tissues, LIPUS therapy will stop. Fortunately, LIPUS stimulated no HO in this study.

## 5. Conclusions

This study reports a feasibility study of fracture nonunion treated with LIPUS and monitored with ultrasonography. The results suggest that ultrasonography is a potential tool to guarantee the performance of LIPUS therapy with monitoring HO formation. Easy to use, the integration of the handheld ultrasound scanner and the ultrasonic therapeutic apparatus is entirely dedicated to help orthopedists make high-quality care and diagnosis.

## Data Availability

All datasets generated for this study are included in the article.
